# Effects of acute caffeine, theanine and tyrosine supplementation on mental and physical performance in athletes

**DOI:** 10.1186/s12970-019-0326-3

**Published:** 2019-11-26

**Authors:** Javier Zaragoza, Grant Tinsley, Stacie Urbina, Katelyn Villa, Emily Santos, Angelie Juaneza, Matthias Tinnin, Cory Davidson, Susan Mitmesser, Zhiying Zhang, Lem Taylor

**Affiliations:** 1 School of Exercise & Sports Science, Human Performance Lab, University of Mary Hardin-Baylor, Belton, TX 76513 USA; 2 Department of Kinesiology & Sport Management, Texas Tech University, Lubbock, TX USA; 3 Guardian Premiere Solutions Special Warfare, San Antonio, TX 78236 USA; 4 Department of Cardiology, Cardiac Rehabilitation, Scott & White Medical Center, Temple, TX 76508 USA; 5 Department of Nutrition & Scientific Affairs, The Nature’s Bounty Co., 2100 Smithtown Ave, Ronkonkoma, NY 11779 USA

**Keywords:** Caffeine, Reaction time, Mental performance, Dietary supplements

## Abstract

**Background:**

A limited amount of research has demonstrated beneficial effects of caffeine and theanine supplementation for enhancement of mental performance. The purpose of this investigation was to determine whether the acute ingestion of a supplement containing caffeine, theanine and tyrosine improves mental and physical performance in athletes.

**Methods:**

Twenty current or former male collegiate athletes (age: 20.5 ± 1.4 y; height: 1.82 ± 0.08 m; weight: 83.9 ± 12.6 kg; body fat: 13.8 ± 5.6%) completed this randomized, double-blind, placebo-controlled crossover trial. After familiarization, each participant completed two identical testing sessions with provision of a proprietary dietary supplement (SUP) containing caffeine theanine and tyrosine or a placebo (PL). Within each testing session, participants completed assessments of mental and physical performance before and after provision of SUP or PL, as well as after two rounds of exercise. Assessments were performed using a performance testing device (Makoto Arena) that evaluated multiple aspects of mental and physical performance in response to auditory and visual stimuli. Testing was performed both with the body in a static position and during dynamic movement. General linear models were used to evaluate the effects of SUP and PL on performance.

**Results:**

Changes in movement accuracy during performance assessment were greater following SUP ingestion as compared to PL for both static and dynamic testing (SUP: + 0.4 to 7.5%; PL: − 1.4 to 1.4% on average; *p* < 0.05). For dynamic testing, the change in number of targets hit was higher and the change in average hit time was lower with SUP as compared to PL (*p* < 0.05). However, there were no differences between conditions for the changes in number of targets hit or average hit time during static testing. There were no differences in changes of subjective variables during either condition, and performance measures during the two rounds of exercise did not differ between conditions (*p* > 0.05).

**Discussion:**

The present results indicate that a combination of a low-dose of caffeine with theanine and tyrosine may improve athletes’ movement accuracy surrounding bouts of exhaustive exercise without altering subjective variables. Based on this finding, supplementation with caffeine, theanine and tyrosine could potentially hold ergogenic value for athletes in sports requiring rapid and accurate movements.

**Trial registration:**

NCT03019523. Registered 24 January 2017.

## Introduction

Compounds that could potentially enhance mental and physical performance could have importance in tasks of daily living and to various athletic populations. When consumed, such compounds could alter focus, attention, and reaction time. As such, it is well known that a variety of compounds found in food and beverages are bioactive and may alter mental and physical performance. Furthermore, many of these compounds are isolated or purified and utilized in dietary supplement formulations [1]. One of the most popular of these substances is caffeine, which is commonly consumed worldwide and is present in a broad array of dietary supplements [2]. Caffeine is known to have stimulatory effects on the central nervous system via antagonistic action on adenosine receptors [3]. Thus, caffeine can increase levels of dopamine, acetylcholine, and serotonin [3]. As such, caffeine is commonly used to suppress feelings of fatigue and increase feelings of energy and concentration.

Furthermore, caffeine consumption has been reported to enhance several aspects of performance at doses of ≥3 mg/kg body weight, including vigilance, reactive agility and physical performance in maximal endurance exercise and high-intensity intermittent exercise [3–5]. It has also been reported that caffeine intake can improve cognitive performance in the context of physical fatigue or sleep deprivation [6–8]. Some data support beneficial effects of lower doses of caffeine (~ 1 mg/kg) on attention, alertness and reaction time [9–11], although limited information is available concerning the effects of low doses of caffeine on both cognitive and physical performance, particularly in athletes or other active individuals.

While supplemental caffeine is sometimes consumed in isolation, naturally-occurring caffeine is found in foods alongside other bioactive ingredients. In tea, one such compound is theanine, a non-proteinogenic amino acid associated with improvements in cognitive function [12]. In isolation, evidence shows theanine engenders decrements in performance or has an absence of effects [13]. However, due to the co-occurrence of caffeine and theanine in tea, and potential complementarity, several studies have evaluated whether caffeine and theanine exhibit synergistic effects for enhancement of cognitive performance or related variables [13–21]. These studies have demonstrated that the combination of caffeine and theanine may produce superior cognitive performance, as compared to either compound individually or a placebo, in some instances [14, 15, 17, 18, 21]. Camfield et al. [20] performed a meta-analysis of randomized placebo-controlled trials and found that the combination of caffeine and theanine exerted favorable effects on alertness and attentional switching accuracy in the first 2 h after ingestion, with standardized mean differences (SMDs) relative to placebo of 0.39 to 0.54 for alertness and SMDs of 0.29 to 0.38 for attention switching accuracy.

Another dietary compound that has been suggested to potentially exert cognition-enhancing effects is the amino acid tyrosine. Tyrosine can be found in protein-rich foods such as dairy, meat, eggs, and nuts [22]. Tyrosine serves as a precursor to the catecholamines dopamine, epinephrine and norepinephrine, and plasma tyrosine has demonstrated a dose-response relationship after ingestion of 100 to 200 mg/kg, with peak plasma concentrations of the compound reached at 2 h post ingestion [22]. A limited amount of research has indicated that supplemental tyrosine may improve working memory updating during cognitively-demanding tasks [23], as well as divergent thinking [24].

While varying amounts of research have examined their effects individually, there is a lack of information concerning the cognitive and physical performance effects of the combination of caffeine, theanine and tyrosine. Additionally, previous studies have not examined the effects of these compounds in athletes, for whom cognitive performance in the context of physical demands is paramount. Due to the possible synergy of these dietary compounds, as well as the lack of information in athletic individuals, the purpose of this investigation was to determine whether the acute ingestion of a supplement containing caffeine, theanine and tyrosine improves cognitive and physical performance in male athletes.

## Methods

### Overview

This study was a randomized, double-blind, placebo-controlled crossover trial. After familiarization, each participant completed two testing sessions that were identical with the exception of the provided supplement. On one occasion, a dietary supplement (SUP) containing proprietary blend of caffeine, theanine and tyrosine was provided. The SUP contained no other ingredients and only those previously listed. During the other testing session, a placebo (PL) containing ~ 2 g of maltodextrin was provided. Within each testing session, participants completed assessments of mental and physical performance before and after ingestion of the supplement, as well as after two rounds of exercise.

### Participants

Participants were recruited from the university student population. In order to be eligible, individuals were required to meet the following inclusion criteria: 1) male between the ages of 18 and 25; 2) current or former collegiate athlete (all NCAA Division 3); 3) non-smoker; 4) healthy and disease-free; 5) regular caffeine consumer; and 6) willingness to comply with study protocol. To quantify caffeine consumption, each participant completed a caffeine questionnaire identifying daily habitual caffeine intake. Additionally, each participant completed a health history questionnaire and the Physical Activity Readiness Questionnaire (PAR-Q). Individuals were ineligible for participation if they: 1) had not been engaged in regular exercise training for at least 12 months prior to the study; 2) answered “yes” to any question on PAR-Q; 3) were diagnosed with any disease or were currently taking prescription medications; 4) were currently using a dietary supplement other than a multi-vitamin/mineral, protein powder or meal replacement, or had used such supplements in the past 6 weeks; 5) were currently enrolled in another research study or had been enrolled in the past 8 weeks; or 6) had any known allergy or sensitivity to caffeine or other stimulants, as determined by the health history questionnaire. Each participant also completed an adverse events questionnaire prior to and at the end of each testing session. Questions included, but were not limited to, symptoms of gastrointestinal distress, headaches, and dizziness. A university-approved informed consent document was signed by each participant prior to study commencement. This study was approved by the Institutional Review Board at the University of Mary Hardin-Baylor.

### Visits

#### Familiarization

After screening and informed consent, the height and weight of each participant was obtained using standard procedures. Body composition of each participant was assessed via multi-frequency bioelectrical impedance analysis (InBody 770, Seoul, Korea). Participants were also familiarized with the performance testing device used in this study (Makoto Arena, Makoto USA, Illinois). This device evaluates reaction time, cognitive function and overall mental and physical performance during specific tasks. This performance testing device consists of three towers, each with lights located at the base as well as at other locations on the tower. During testing, audio is associated with flashing lights with higher pitch noises corresponding to light targets at a higher vertical placement on the tower and lower pitch noises corresponding to targets at the base of the towers. Lights on the left side of the towers were required to be hit by the participant’s left hand, while lights on the right side of the tower were required to be hit by the participant’s right hand and lights in the middle of the tower could be hit with either hand. Lights at the base of the tower were required to be hit by the participant’s feet. During one-tower testing, participants remained adjacent to a single tower during the 30-s test, which was performed on level 11 (i.e. the fastest setting). During three-tower testing, participants were required to move about the arena in order to hit lights produced by any of the three towers. The three-tower testing was performed on level 6 (i.e. a mid-range speed). The specific variables assessed by the performance testing system were the total targets, targets hit, average hit time and accuracy. During familiarization, participants completed trials on the device until they had less than a 10% difference between consecutive scores for targets hit. On average, this took approximately 6 trials. Following familiarization, participants were randomly assigned to an order in which to complete the two testing sessions.

#### Visit 1

Participants provided dietary records for the 3 days prior to each testing session. Additionally, they were required to abstain from unfamiliar exercise and exercise of greater-than-usual intensity for 48 h prior to each session, as well as abstain from all exercise for 24 h prior to each session. Each participant was allowed an ad libitum breakfast on the day of testing, followed by a standardized lunch (MET-Rx® Big 100 meal replacement bar; 400 kcal, 48 g carbohydrate, 10 g fat, 31 g protein) that was consumed 2 to 3 h prior to the acute testing session. Water was the only beverage allowed on the day of testing, and participants were instructed to discontinue water consumption one hour prior to testing. Caffeine intake was disallowed 24 h prior to and on the day of testing. At the beginning of each session, pre-supplementation (T1) assessments took place. These included evaluations of body weight, resting hemodynamic variables (i.e. heart rate and blood pressure), standard visual analog scales (VAS) to quantify subjective variables (i.e. energy, focus, concentration, alertness, fatigue and motivation) and testing on the Makoto performance assessment device. After these tests were completed, each participant ingested the assigned dietary supplement (i.e. SUP or PL) with 8 oz of water under researcher supervision then rested quietly for 30 min. Following this rest period, the VAS and Makoto assessments were repeated (T2) before each participant completed one round of exercise. The round of exercise consisted of nine individual exercises, each of which were performed continuously for 45 s with 10 s of rest between exercises. The individual exercises included were: small battle rope waves, large battle rope waves, battle rope slams, kettlebell swings, line jumps, toe touches, mountain climbers, bosu squats and burpees. Physical performance during each round of exercise was quantified via the amount of reps completed per exercise per set, and heart rate was monitored before and after each round of exercise and Makoto assessment. Upon completion of the first round of exercise, the VAS and Makoto assessments were repeated (T3), with the Makoto assessments commencing 90 s after the completion of the exercise round. This was followed by a second identical round of exercise and final VAS and Makoto assessments (T4). At the end of the final one- and three-tower Makoto assessments, the Makoto infinity test was performed. This testing program continuously generated a random sequence of visual targets and auditory signals which prompted the participant to hit the specified targets. The infinity test continued until the participant missed 3 total targets. In addition to the performance testing procedures, each participant completed a side effects questionnaire.

#### Visit 2

Following the completion of the first condition, participants entered a 1-week washout period with instructions to maintain habitual exercise and nutritional routines. Participants adhered to the same pre-assessment procedures and completed an additional 3-day diet record prior to the second condition. The second condition was identical to the first, with the exception of which dietary supplement was provided. Additionally, the second condition occurred on the same day of the week and time as the first condition. For both conditions, participants were not scheduled on “high stress” days, including those with examinations or athletic competitions.

### Statistical analysis

General linear models were used to test for the effects of the dietary supplement condition, time, and their interactions on mental and physical performance. Data were transformed when skewed distributions were present. Change scores, where applicable, were generated by subtracting the baseline values from the values at each subsequent time point (i.e. T2, T3 and T4). Non-parametric Kruskal-Wallis tests were conducted for measures with skewed distribution to compare the differences cross treatment groups at each time point. Paired samples t-tests were used to compare dietary intake, heart rate and blood pressure prior to each session, and general linear models were used to evaluate heart rate responses to exercise. Statistical significance was set at *P* < 0.05. Analyses were performed using Minitab 17 (Minitab Inc., State Collage, PA) and SPSS 25 (IBM, Armonk, NY).

## Results

Twenty participants (age: 20.5 ± 1.4 y; height: 182 ± 8.6 cm; weight: 83.9 ± 12.6 kg; body fat: 13.8 ± 5.6%; average caffeine intake: 263 ± 116 mg/d) completed both conditions and were included in the analysis. There were no differences in dietary intake during the 3 days prior to each condition (calories: *p* = 0.26; carbohydrate: *p* = 0.16; fat: *p* = 0.51; protein: *p* = 0.53).

There were no differences in changes of subjective variables during either testing session, although most variables changed over time in both groups (Table 1). Similarly, there were no differences in exercise performance during either condition (Table 2). Heart rate (*p* = 0.51), systolic blood pressure (*p* = 0.34) and diastolic blood pressure (*p* = 0.77) measured at the beginning of each visit did not differ between conditions. Additionally, heart rate responses to the exercise rounds did not differ (*p* = 0.53 for condition by time interaction). Heart rate increased in both conditions during each exercise round (round 1: + 45 to 50 bpm; round 2: + 26 to 31 bpm; *p* < 0.001 for time main effect). No side effects of supplement consumption were reported during either condition.

**Table 1:** Subjective variables from the VAS scale

	Condition	Δ (T2-T1)	Δ (T3-T1)	Δ (T4-T1)	*p* (group)	*p* (time)	*p* (interaction)
Energy	PL	0.77 ± 0.21	−1.56 ± 0.37	−2.25 ± 0.49	0.33	< 0.001*	0.85
	SUP	0.49 ± 0.17	− 1.64 ± 0.28	− 2.72 ± 0.43			
Focus	PL	0.61 ± 0.24	− 0.93 ± 0.30	−1.64 ± 0.43	0.31	< 0.001*	0.89
	SUP	0.49 ± 0.19	−1.25 ± 0.39	−2.12 ± 0.55			
Concentration	PL	0.59 ± 0.25	−0.82 ± 0.33	− 1.54 ± 0.47	0.19	< 0.001*	0.69
	SUP	0.56 ± 0.18	−1.33 ± 0.30	− 2.14 ± 0.49			
Alertness	PL	0.58 ± 0.29	− 0.80 ± 0.27	− 1.33 ± 0.45	0.53	< 0.001*	0.76
	SUP	0.59 ± 0.26	−0.87 ± 0.30	− 1.80 ± 0.49			
Fatigue	PL	−0.10 ± 0.19	1.04 ± 0.44	1.24 ± 0.76	0.92	0.09	0.96
	SUP	0.06 ± 0.50	0.89 ± 0.55	1.10 ± 0.77			
Motivation	PL	0.27 ± 0.23	−1.15 ± 0.35	−2.52 ± 0.52	0.71	< 0.001*	0.89
	SUP	0.49 ± 0.22	−1.25 ± 0.39	−2.28 ± 0.50			

Data displayed as mean ± SE

*PL* placebo, *SUP* supplement containing caffeine, theanine and tyrosine

*statistically significant time main effect

**Table 2:** Quantification of exercise performance variables

	Condition	Exercise round 1	Exercise round 2	*p* (group)	*p* (time)	*p* (interaction)
Small Battle Rope	PL	80.6 ± 18.9	77.6 ± 16.2	0.99	0.10	0.31
	SUP	85.4 ± 27.7	72.7 ± 20.6			
Large Battle Rope	PL	49.1 ± 6.7	43.9 ± 7.8	0.11	0.04*	0.84
	SUP	45.0 ± 12	40.8 ± 12			
Battle Rope Slams	PL	20.5 ± 4.4	19.4 ± 4.7	0.72	0.21	0.84
	SUP	20.3 ± 3.6	18.9 ± 4.9			
Kettlebell Swings	PL	22.1 ± 3.1	21.2 ± 3.1	0.87	0.09	0.81
	SUP	22.6 ± 2.9	21.5 ± 3.4			
Line Jumps	PL	118.4 ± 12.1	115.7 ± 14.9	0.65	0.89	0.56
	SUP	114.5 ± 16.7	116.2 ± 21.8			
Toe Touches	PL	130.0 ± 28.2	119.0 ± 19	0.32	0.14	0.68
	SUP	121.9 ± 26.6	115.7 ± 27.2			
Mountain Climbers	PL	92.8 ± 17.9	88.5 ± 23.1	0.84	0.48	0.87
	SUP	91 ± 23.2	88.3 ± 25			
Bosu Squats	PL	17.6 ± 5.5	18.6 ± 6.1	0.26	0.70	0.84
	SUP	19.8 ± 9.5	20.1 ± 8.2			
Burpees	PL	13.6 ± 1.8	12.6 ± 2.6	0.83	0.35	0.45
	SUP	13.3 ± 2.3	13.2 ± 3.1			

The data displayed shows how many repetitions were completed for each variable during the first round of exercise and the second round of exercise. Data displayed as mean ± SD

*PL* placebo, *SUP* supplement containing caffeine, theanine and tyrosine

*statistically significant time main effect

For the one tower (i.e. static) Makoto testing, a group main effect was present indicating higher changes in accuracy with SUP across the three post-supplementation assessments. At the final assessment, PL demonstrated a decrease of 1.4% from baseline while SUP demonstrated an increase of 3.1% (Table 3). However, there were no differences between conditions for targets hit or average hit time. For the three tower (i.e. dynamic) Makoto testing, group main effects were present for changes in targets hit, average hit time and accuracy. At the three post-supplementation time points, the number of targets hit in SUP was 1.6 to 3.5 higher than the pre-supplementation time point (i.e. T1), while the number of targets hit in PL was 0.4 lower to 0.4 higher than at T1. Accompanying this difference were improved accuracy changes with SUP relative to PL from T1 to the three post-supplementation time points (PL: − 0.4 to + 1.4%; SUP: + 3.7 to 7.5%). Additionally, at T3 and T4, average hit time was 0.004 to 0.01 s lower than baseline in SUP, while average hit time was 0.004 to 0.006 s higher than baseline in PL. Total targets did not differ between conditions for either one tower or three tower testing. For the Makoto infinity testing, there were no differences between conditions for total targets (*p* = 0.28), targets hit (*p* = 0.29), average hit time (*p* = 0.71) or accuracy (*p* = 0.26).

**Table 3:** Makoto performance testing

		Condition	Δ (T2-T1)	Δ (T3-T1)	Δ (T4-T1)	*p* (group)	*p* (time)	*p* (interaction)
One-tower Testing	Total Targets (#)	PL	−0.20 ± 0.24	−0.20 ± 0.24	− 0.30 ± 0.19	0.18	0.74	0.90
		SUP	− 0.55 ± 0.25	− 0.35 ± 0.31	− 0.65 ± 0.30			
	Targets Hit (#)	PL	− 0.25 ± 0.49	0.10 ± 0.42	−0.7 ± 0.39	0.52	0.66	0.33
		SUP	−0.45 ± 0.48	0.00 ± 0.45	0.30 ± 0.42			
	Average Hit Time (s)	PL	0.0005 ± 0.008	−0.0005 ± 0.008	0.002 ± 0.008	0.40	0.67	0.70
		SUP	0.003 ± 0.008	−0.01 ± 0.009	−0.008 ± 0.011			
	Accuracy (%)	PL	−0.25 ± 1.07	0.90 ± 0.85	−1.35 ± 1.08^a^	0.03*	0.54	0.07
		SUP	0.35 ± 1.07	1.25 ± 0.94	3.10 ± 0.84^a^			
Three-tower Testing	Total Targets (#)	PL	0.05 ± 0.19	−0.40 ± 0.20	−0.30 ± 0.21	0.75	0.30	0.84
		SUP	0.16 ± 0.38	0.05 ± 0.36	−0.26 ± 0.36			
	Targets Hit (#)	PL	−0.10 ± 0.97^a^	0.35 ± 1.02	−0.40 ± 0.76	0.003*	0.51	0.79
		SUP	3.47 ± 1.10^a^	3.26 ± 1.29	1.63 ± 1.53			
	Average Hit Time (s)	PL	−0.002 ± 0.008	0.006 ± 0.010	0.004 ± 0.009	0.044*	0.92	0.62
		SUP	−0.006 ± 0.006	−0.01 ± 0.007	−0.004 ± 0.008			
	Accuracy (%)	PL	−0.45 ± 1.95^a^	1.40 ± 2.01	−0.20 ± 1.56	0.004*	0.53	0.80
		SUP	6.63 ± 2.24^a^	7.53 ± 2.90	3.74 ± 3.33			

Data displayed as mean ± SE

*PL* placebo, *SUP* supplement containing caffeine, theanine and tyrosine

*statistically significant group main effect

^a^significant difference between PL and SUP at this time point

## Discussion

The purpose of this investigation was to determine whether the acute ingestion of a supplement containing caffeine, theanine and tyrosine improves cognitive and physical performance in male athletes. The primary findings were that the dietary supplement improved some aspects of performance during tasks involving both cognitive and physical demands, without affecting subjective mental states or exercise performance during exhausting bouts of exercise. While accuracy was improved with supplementation for both static (*p* = 0.026) and dynamic testing (*p* = 0.004), the supplement appeared to produce greater effects during dynamic testing. In static testing, when compared to placebo, greatest improvements of accuracy were seen at the third time point (*p* = 0.002). Furthermore, larger increases in accuracy, greater reductions in average hit time and greater increases in the number of targets hit were observed with supplementation during dynamic testing conditions Reductions in average hit time ranged from 4 milliseconds (ms) faster when compared to baseline to 11 ms. Although these reductions in average hit time were not statistically significantly different at each individual time point, there was a treatment effect (*p = 0.044)*. Taken together, these results may indicate the potential for ergogenic effects of caffeine, theanine and tyrosine in athletes whose sports require rapid and accurate responses during body movement.

The dietary supplement examined in the present study contained caffeine, theanine and tyrosine. The dose of caffeine, which equated to approximately 1 mg/kg body weight, was substantially lower than the dose of caffeine typically associated with ergogenic effects on endurance and high-intensity exercise performance (i.e. 3 to 6 mg/kg) [3]. Although sensitivity to caffeine varies between individuals [25], some report unwanted side effects, such as restlessness, nervousness and agitation, with ingestion of moderate to high doses [26]. Athletes who consume caffeine for ergogenic purposes, but who do not wish to experience altered mental states or side effects that could potentially be detrimental to performance, could potentially benefit from lower doses of caffeine. The present study supports improvement of some aspects of mental and physical performance, without alteration of subjective feelings of energy, focus, concentration and related parameters. This could be a potentially desirable outcome for trained athletes who want to maximize cognitive performance without altering their mental state during training or competition. Due to the proprietary nature of the supplement (e.g. exact concentrations of each ingredient are not released), it should again be noted that such outcomes could be due to the combination of caffeine, theanine and tyrosine, and not specifically due to the caffeine alone. While crude estimates of exercise performance (i.e. repetitions performed during timed exercise) were not improved by supplementation in the present study, the rounds of exercise were not sport-specific and were intended to produce fatigue in order to determine the effects of the dietary supplement in these conditions. As such, performance during the Makoto assessments are likely more indicative of whether caffeine, theanine and tyrosine could improve performance in activities requiring rapid mental and physical responses and movement accuracy.

The potential for synergy between caffeine and theanine, for the purpose of enhancement of cognitive performance, has previously been examined with mixed results [13–21]. Haskell et al. noted decreases in performance of various mental tasks paired with increased headache ratings in theanine treatment alone. However, when paired with caffeine, performance in mental tasks improved along with mental state parameters such as feelings of fatigue and alertness [14]. Contrary to previous investigations of theanine and caffeine, hemodynamic measures did not differ between treatments in the current investigation [16]. Despite this, no alterations in subjective mental states were present in the current investigation suggesting the added effects of theanine on mental states. Studies demonstrating potential cognitive-enhancing effects of the combination have used doses of 40 to 160 mg of caffeine and 97 to 250 mg of theanine [14, 15, 17, 18, 21]. The doses of these compounds used in the present study were similar, although the dose of theanine was slightly lower than previous investigations. To our knowledge, the addition of tyrosine to caffeine and theanine has not previously been examined for the enhancement of cognitive and physical performance in athletes. However, a single study investigated the effects of a nutrient enriched breakfast bar containing, but not limited to, caffeine, theanine, and tyrosine in healthy adults over 8 weeks with no exercise intervention [27]. The authors suggested the synergistic effects of caffeine, theanine, and tyrosine were the ingredients involved in improving performance of various mental tasks. Doses, although similar to the current investigation, were lower in caffeine and tyrosine [27]. The dose of tyrosine in the present investigation is similar to the dose used in two previous investigations demonstrating potential benefits for some aspects of cognitive function [23, 24]. Despite these findings, the additive and synergistic effects of the compounds in question was not investigated in the present study.

The mechanisms underlying the potential synergistic effects of caffeine and theanine are not known. Neurochemically, both caffeine and theanine have fostered changes in neurotransmitter systems. Such targets include dopamine, serotonin, and glutamate [28]. Despite this, and to the knowledge of the authors, no investigation has proceeded looking at the combined effects of caffeine and theanine at the receptor level. One recent investigation outlined findings that were focused on the neuroprotective effects of these compounds and identified the antagonistic effect theanine has on glutamate receptors as the primary driver through which theanine elicits positive benefits [28]. As previously mentioned, there is a lack of knowledge concerning the cognitive and physical performance effects of the combination of caffeine, theanine and tyrosine. Thus, the effects of these three compounds, in combination, at the receptor level remain elucidated. However, while the results of the present study are promising, additional research of the individual and additive effects of these dietary compounds is warranted. As such, it should be considered that the current study has limitations. Firstly, there were only two groups (placebo vs. active) with no other groups testing the compounds in isolation (theanine only, etc.). As such, the results could be due to any one of the compounds either in combination or isolation, and not due to the low-dose of caffeine plus theanine and tyrosine. Secondly, plasma levels of caffeine or other levels of the compounds were not evaluated. It should be dually noted that the individual variability of caffeine tolerance plus the potential for changes in caffeine pharmacokinetics with coingestion of theanine and tyrosine could have played a role in timing of testing.

## Conclusion

In conclusion, the present results suggest that a combination of a low-dose of caffeine with theanine and tyrosine may improve athletes’ movement accuracy surrounding bouts of exhaustive exercise without altering subjective mental states. Based on this finding, supplementation with caffeine, theanine and tyrosine could potentially hold ergogenic value for athletes in sports requiring rapid accurate movements.

## Data Availability

The datasets used and/or analyzed during the current study are available from the corresponding author on reasonable request.

## Abbreviations

ms: Milliseconds
PAR-Q: Physical Activity Readiness Questionnaire
PL: Placebo
SMDs: Standardized mean differences
SUP: Supplement
VAS: Visual analog scales
